# Supplementary figures and images for: The Arabidopsis receptor kinase STRUBBELIG regulates the response to cellulose deficiency
Source: PLoS Genet. 2020 Jan 21;16(1):e1008433. doi: 10.1371/journal.pgen.1008433 (PMC6994178; doi:10.1371/journal.pgen.1008433)

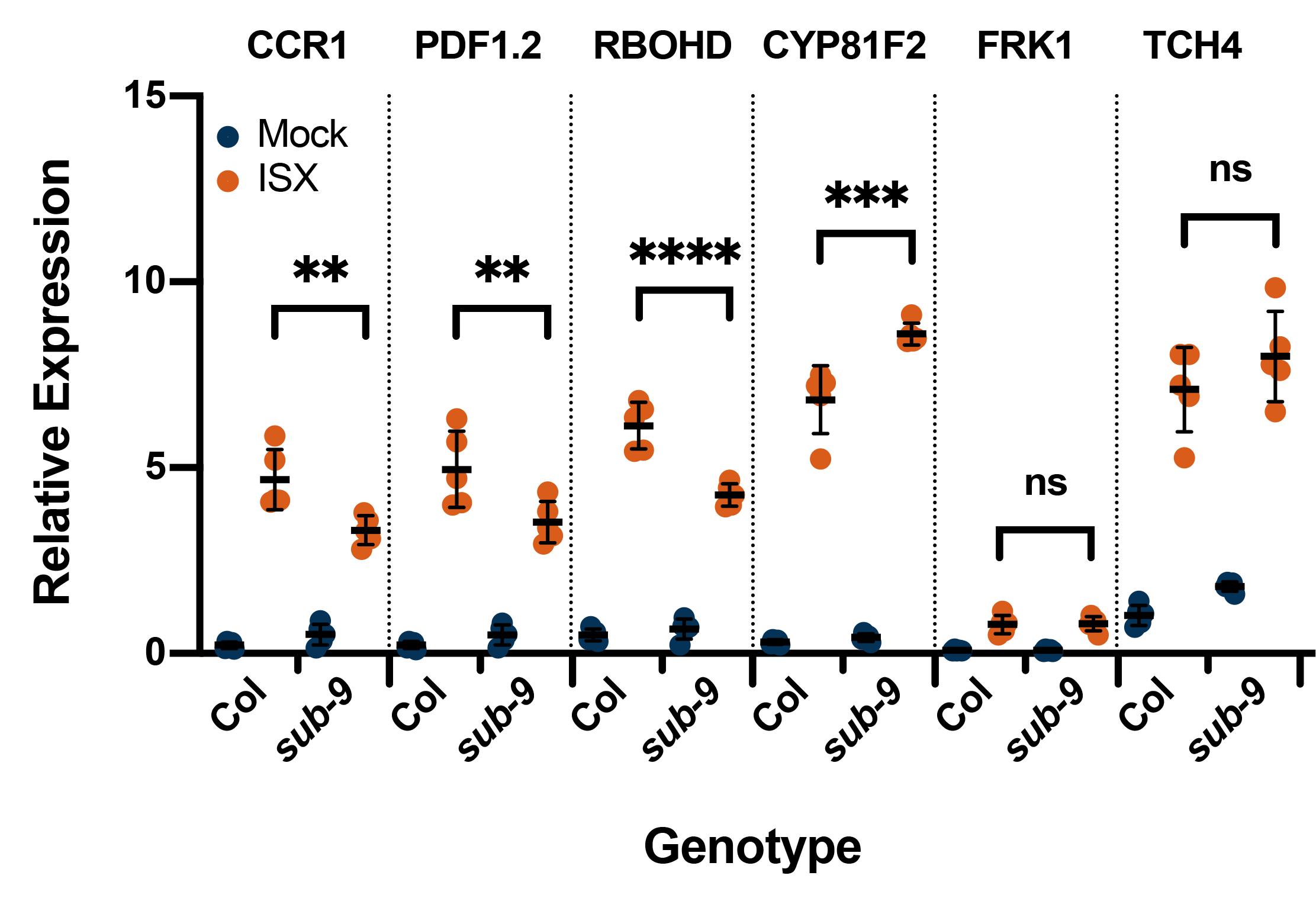

Supplement: S1 Fig — Gene expression levels of several CBI marker genes by qPCR upon exposure of seven-day-old seedlings to 600 nM isoxaben for eight hours. The results from five biological replicates are shown. Marker genes and genotypes are indicated. Mean ± SD is presented. Asterisks represent adjusted P values (** P < 0.009, *** P = 0.0002, **** P < 0.0001, ns: not significant; one-way ANOVA followed by Tukey’s multiple comparison tests). For all genotypes the differences between isoxaben and mock treatments were statistically significant (adjusted P value < 0.0001). Differences between mock treated wild-type and sub-9 samples were not statistically significant. The experiment was repeated twice with similar results. (TIF) [file pgen.1008433.s001.tif]

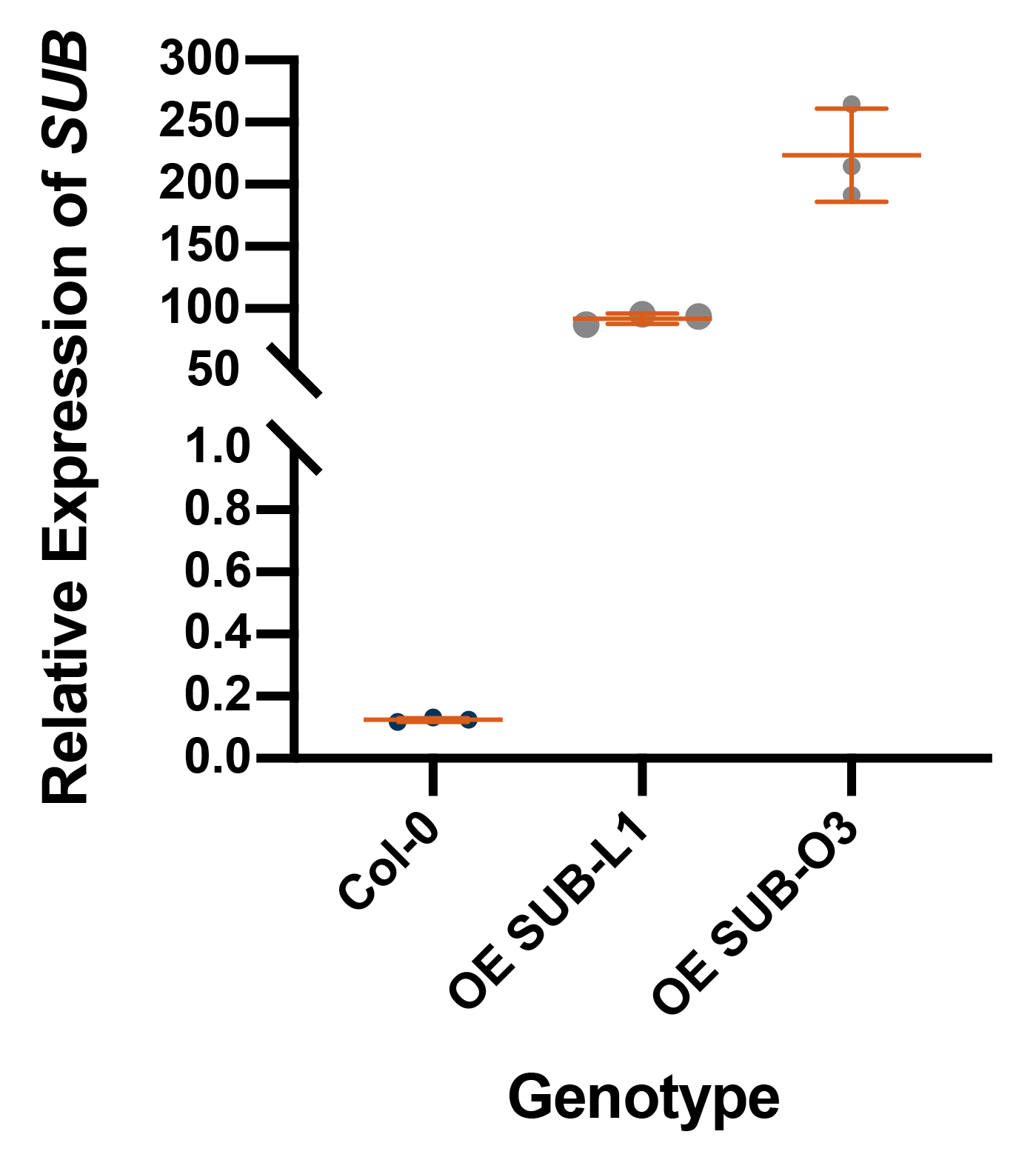

Supplement: S2 Fig — The results from three biological replicates are shown. Mean ± SD is presented. (TIF) [file pgen.1008433.s002.tif]

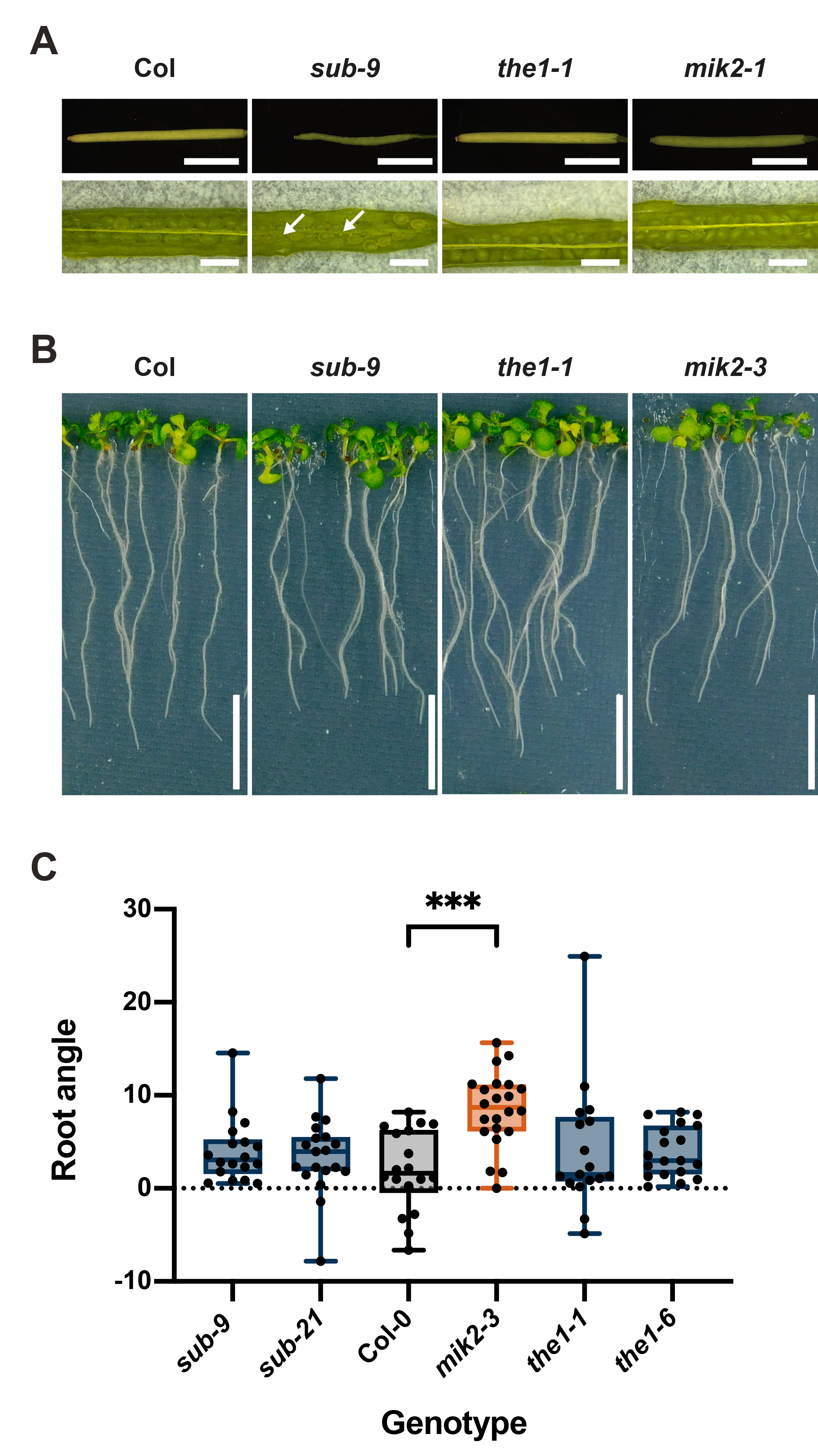

Supplement: S3 Fig — (A) Upper panel: siliques. Bottom panel: open siliques revealing developing seeds. Genotypes are indicated. Note aberrant sub-9 silique morphology and the reduced number of developing seeds in sub-9. Arrows indicate undeveloped seeds. (B) Root angle of nine-day-old seedlings grown in an upright position (10° angle relative to direction of gravity) on half-strength MS agar medium supplemented with 1% sucrose. Pictures were taken from the front of the plate. Genotypes are indicated. Note the slight slant to the left in mik2-3. (C) Quantification of results depicted in (B) and as described in Van der Does et al (2017) PLoS Genet 13: e1006832. Genotypes are indicated. Box and whisker plots are shown. Whisker ends mark the minimum and maximum of all the data. 16 ≤ n ≤ 22. Asterisks represent adjusted P values (*** P = 0.0001; one-way ANOVA followed by Tukey’s multiple comparison tests). The experiment was performed twice. In the second experiment the root angle difference in mik2-3 was not statistically significant. Scale bars: open siliques, 1 mm; closed siliques, 0.5 cm; roots, 1 cm. (TIF) [file pgen.1008433.s003.tif]
