# Supplementary material for: The Arabidopsis receptor kinase STRUBBELIG regulates the response to cellulose deficiency
Source: PLoS Genet. 2020 Jan 21;16(1):e1008433. doi: 10.1371/journal.pgen.1008433 (PMC6994178; doi:10.1371/journal.pgen.1008433)
Supplement: S1 Table — (DOCX) [file pgen.1008433.s004.docx]

**S1 Table. Primers used in this study.**

| **Primer name** | **Sequence** |
| --- | --- |
| R1(at4g33380) LP | 5’- TGAAGGAGAGGAAGAGCCTGAGGAA -3’ |
| R1(at4g33380) RP | 5’-CCCCATCTCACTGCAGCACCAC -3’ |
| R2 At2g28390 LP | 5’-AGATTGCAGGGTACGCCTTGAGG-3’ |
| R2 At2g28390 RP | 5’- ACACGCATTCCACCTTCCGCG -3’ |
| R3 At5g46630 LP | 5’- CCAAATGGAATTTCAGGTGCCAATG -3’ |
| R3 At5g46630 RP | 5’- CAATGCGTACCTTGAGAAAACGAAC-‘3 |
| *CCR1*(At1G15950) LP | 5’-GTTCTCTTTGCTGCTTTCGAC-3’ |
| *CCR1*(At1G15950) RP | 5’-GCAAACCCCTGACCATGT-3’ |
| *CCR2*(At1G80820) LP | 5’-GTTCTCTTTGCTGCTTTCGAC-3’ |
| *CCR2*(At1G80820) RP | 5’-GCAAACCCCTGACCATGT-3’ |
| *PDF1.2*(AT5G44420) LP | 5’- TCTTTGCTGCTTTCGACGC-3’ |
| *PDF1.2*(AT5G44420) RP | 5’- TCTTGCATGCATTACTGTTTCCG-3’ |
| *VSP1* (AT5G24780) LP | 5’- GATATGGGACCGAGAACACAGC-3’ |
| *VSP1*(AT5G24780) RP | 5’ TTCGTATAGATGCAAGGTCTCCG-3’ |
| *FRK1* (At2g19190) LP | 5’-ATCTTCGCTTGGAGCTTCTC-3’ |
| *FRK1* (At2g19190) RP | 5’-TGCAGCGCAAGGACTAGAG-3’ |
| *CYP81F2* (At5g57220) LP | 5’-AATGGAGAGAGCAACACAATG-3’ |
| *CYP81F2* (At5g57220) RP | 5’-ATACTGAGCATGAGCCCTTTG-3’ |
| *RBOHD* (AT5G47910) LP | 5′-CTGCTCCGTGCTTTCAGAT-3′ |
| *RBOHD* (AT5G47910) RP | 5′-AATCCTTGTGGCTTCGTCAT-3′ |
| *TCH4* (AT5G57560) LP | 5’-GATCACTTGGGGTGATGGTC-3’ |
| *TCH4* (AT5G57560) RP | 5’-GGGACAAGCTTCATTTGCAT-3’ |
| *TIP2;3* (AT5G47450) LP | 5’-GAAGTTGGAAGTGTGGGAGACT-3’ |
| *TIP2;3* (AT5G47450) RP | 5’-GCTCCATCAGAGGTTAGTTTGG-3’ |
| *CesA1*(At4g32410) LP | 5’-GATCCGACATGAATCTGATGG-3’ |
| *CesA1*(At4g32410) RP | 5’-CACATTCATTACACGCGACA-3’ |
| *CesA3*(At5g05170) LP | 5’-GTCAGATTGGGGAATGGAGA-3’ |
| *CesA3*(At5g05170) RP | 5’-GCTTAGGCATGCAGTAAATGG-3’ |
| *CesA6*(At5g64740) LP | 5’-ACCCGGATTTGATCACCATA-3’ |
| *CesA6*(At5g64740) RP | 5’-GAACCCCAGAGACTCGTATCA-3’ |
| CesA2(At4G39350) LP | 5’-CGCTAGAGAATGTCGACGAA-3’ |
| CesA2(At4G39350) RP | 5’-TAGGAATGCGAGTCCAGCTT-3’ |
| CesA5(AT5G09870) LP | 5’-CCGTAGATCCACCCAATCTC-3’ |
| CesA5(AT5G09870) RP | 5’-GAAAATTCATCGTCCCTGAGA-3’ |
| CesA9(AT2G21770) LP | 5’-CGTTACCGCAATGGACATAA-3’ |
| CesA9(At2G21770) RP | 5’-GTTCTCTTTGCTGCTTTCGAC-3’ |
| *SUB*(AT1G11130) LP | 5’- GTTTGGATCTTTGACCTAGACGA-3’ |
| *SUB*(AT1G11130) RP | 5’- CAAGTTATTAATCGCCGAAACAT-3’ |
| *SUBgRNA* | 5’ TAATAACTTGTATATCAACTT-3’ |
